# Considerations for establishment of a private virtual hospital identified using an implementation science approach

**DOI:** 10.1038/s41598-025-85965-5

**Published:** 2025-01-29

**Authors:** Olivia J. Fisher, Caroline Grogan, Andrew Barron, Shanthi Kanagarajah, Sue-Ellen Smith, Ian Smith, Kelly McGrath

**Affiliations:** 1 Wesley Research Institute, Brisbane, Australia; 2 Faculty of Health, Charles Darwin University, Darwin, Australia; 3 Faculty of Health, The University of Queensland, Brisbane, Australia; 4 UnitingCare Queensland, Brisbane, Australia; 5 Queensland Physician Care, Brisbane, Australia; 6 BlueCare Community, Brisbane, Australia; 7 St Andrew’s War Memorial Hospital, Spring Hill, Australia

**Keywords:** Healthcare delivery, Implementation science, Virtual hospital, Virtual healthcare, Digital health, Health services research, Health services, Health occupations

## Abstract

Virtual hospitals are rapidly being implemented internationally. Research has predominantly focused on clinical outcomes not implementation. We aimed to identify pre-implementation determinants to enable health services to tailor virtual hospital models, increasing likelihood of suitability, acceptability, uptake, clinical effectiveness, and sustainability. We aimed to inform the design and implementation of a private Australian virtual hospital by identifying contextual barriers, enablers, and considerations. We conducted a qualitative pre-implementation determinant study using snowball sampling and semi-structured interviews (*n* = 37) between February and July 2023 with consumers/carers/both (*n* = 11), clinicians (doctors/allied health/nursing/personal carers), hospital, health service and aged care leadership (*n* = 22), and public health stakeholders (n=4). Deductive framework analysis based on the PERCS implementation science framework was used. The following key determinants were identified: Enablers – strong executive leadership support; enthusiasm for expanding rural and remote services; need for a clear vision; strong tension for change; commitment to high-quality healthcare. Major barrier: restrictive funding models that stifle opportunities for innovation. Other barriers: technological limitations; communication challenges; workforce issues; clinicians’ opinions varied on safety and suitability of virtual healthcare. This implementation science approach enabled identification of a broad set of determinants not previously reported, relevant locally and for an international audience. Evaluation of implementation outcomes is necessary.

## Introduction

The rapid uptake of telehealth and remote monitoring services during the COVID-19 pandemic has seen increased demand for, and acceptance of, virtual healthcare from both patients and providers^1–3^. As evidence for virtual healthcare has grown, there has been a push to expand from small-scale, narrowly-focused telehealth services into full-scale virtual hospitals offering a broad range of services normally provided in a physical hospital setting via telehealth or hybrid clinical services (telehealth with face-to-face and/or monitoring devices)^3–5^. Virtual hospitals are being rolled out internationally, offering hospital-equivalent healthcare in a patient’s home or community, however most have been implemented and adapted ‘on the fly’^5–8^. There is substantial evidence that well designed and delivered virtual hospitals have equal or better clinical outcomes to physical hospitals, low mortality rates, reduced readmissions, cost savings, and high patient satisfaction^1,3,5,7,9–11^. However, as argued in a recent Cochrane Library editorial, research informing design, implementation, scaling and sustainability of virtual hospitals in a broad range of settings is critically absent^12,13^. There is substantial variety in the scope, design and operation of virtual hospitals^3^, and this, when coupled with a lack of high-quality empirical evidence on the most effective component/s of each model, makes designing an evidence-based virtual hospital challenging.

For the purposes of this study we have used the following definitions: ‘telehealth’ – a consultation with a health professional via phone or video conference; ‘hybrid clinical services’ – a combination of telehealth and either remote monitoring devices or a local clinician such as a nurse; ‘virtual hospitals’ – any hospital model without walls, including virtual hospital wards, virtual emergency departments, and hospitals at home. For healthcare organisations considering establishing a virtual hospital, implementation studies are critically important to guide decision making. Although studies have investigated barriers and enablers to virtual hospitals, there are notable research gaps and methodological issues such as: very few pre-implementation studies, a lack of best-practice implementation science methods, and little representation of private hospitals. Ten scoping review, systematic review, or metasynthesis papers were identified: two on virtual hospitals or wards^1,3^, six on hospital in the home^5,11,12,14–16^, one on remote monitoring^17^ and one synthesis of evidence on virtual wards, hospital at home and remote monitoring^10^. In Wallis et al.’s^12^ review of hospital in the home implementation research only six post-implementation and zero pre-implementation studies were identified that used an implementation science model or framework^7,18–22^. The Planning and Evaluating Remote Consultation Services (PERCS) Framework^23^, arguably the most suitable for this purpose, was notably absent from these. Of the few pre-implementation studies identified^24–26^ a narrow set of barriers and enablers were described, primarily focusing on organisational, interventional, inter- and intrapersonal factors. Understanding of context is at the heart of implementation research. There is a paucity of virtual hospital research in the private sector and there is therefore limited ability to generalise findings from much of the existing research to the development of a private virtual hospital. Although the delivery of high-quality healthcare and good patient health outcomes are a priority regardless of sector, there are substantial differences between the key stakeholders, funding models, organisational priorities, and the relationships between doctors and hospitals, as outlined in greater detail in the setting section below. Using an implementation science framework can improve the generalisability of findings as the international and cross-sectoral evidence-base increases and the similarities and differences between sectors, countries and other contexts are better understood.

There have been several notable Australian studies on public virtual hospitals. Shaw et al. reflected on lessons learned following implementation of rpavirtual^8,27^, a public virtual hospital based in Sydney Local Health District. Their post-hoc evaluation report described enablers to implementation: comprehensive clinical governance structures, strong leadership support, strong pre-existing relationships, flexible organisational structure, workforce of clinicians willing to move into new roles, and a strong tension for change to virtual healthcare delivery due to the pandemic. Barriers were: the need to rapidly deviate from the original plan, uncertainty due to frequently changing pandemic guidelines, issues with funding and classification of activity, rapid implementation of new technology platforms and processes creating challenges with legacy systems, need to rapidly upskill a new temporary workforce, and branding confusion. Many of the barriers and enablers identified were specific to rpavirtual, with limited generalisability to other virtual hospitals. In South Australia, My Home Hospital has been established using a hospital in the home model^28^. The patient journey and principles were co-designed with consumers, clinicians, and a broad range of stakeholders. Schultz et al.’s^29^ evaluation of patient experiences of My Home Hospital identified that although patients felt they may have experienced higher quality care in a traditional face-to-face hospital, this was outweighed by the benefits in all other aspects of their lives from being cared for at home. In Queensland, Australia, a virtual ward^7^ and a virtual emergency department^30^ were established by Queensland Health, the statewide public health service, during the COVID-19 pandemic. These services have now been incorporated into the Queensland Virtual Hospital. Evaluation of these services identified that there were equivalent clinical and patient safety outcomes to traditional hospital care, and over time acceptance and adoption were high. In all of these examples, no implementation science theories, models or frameworks were mentioned by the authors, and a limited range of implementation outcomes were discussed, primarily focusing on uptake and acceptance of virtual healthcare.

### Implementation science approach

Implementation science theories, models and frameworks enable healthcare decision makers and clinicians to plan, implement, evaluate and sustain healthcare innovations^31,32^. Using these evidence-based implementation science approaches improves the ability to generalise across settings and contexts by providing a pre-determined set of constructs based on implementation theory that allow comparison between studies. The PERCS Framework was developed by Greenhalgh et al. in the context of rapidly expanding remote public health services during the COVID-19 pandemic^23^. It expanded on previous theoretical approaches and frameworks including the Non-adoption, Abandonment, Scale-up, Spread and Sustainability framework and addressed a gap in the literature at the time on the implementation of remote consultation services^33^. The PERCS Framework provides information about complex stakeholder groups, a set of constructs known to influence remote consultation, and guidance on ethical principles thus making it an appropriate guide to inform virtual hospital design. The theory of organisational readiness for change describes potential individual, group and organisational influences on effective change implementation^34^. It conceptualises organisational readiness as a shared psychological state in which colleagues feel motivated and confident to conduct collective change. Used together with the PERCS Framework, this multi-theoretical approach allowed for a nuanced interpretation of these data.

In this study we used an evidence-based implementation science approach to identify contextual determinants, barriers and enablers to establishment of an Australian private virtual hospital, with the aim of maximising both clinical and implementation effectiveness. This is the first study internationally to use a best-practice evidence-based implementation science approach using the PERCS Framework to identify a comprehensive set of barriers and enablers to establishment of a private virtual hospital. This pre-implementation research can be used as a guide for healthcare decision makers about important considerations and improve planning and delivery of virtual hospitals.

## Methods

This study employed a qualitative pre-implementation design to inform the establishment of a private Australian virtual hospital. This study was conducted in conjunction with a large, private, non-profit, church-led hospital, healthcare, aged care, family and disability service provider in Queensland, Australia (“the health service”). The health service aimed to implement a private virtual hospital and engaged the research team in September 2022 to conduct a context assessment and provide recommendations on barriers and enablers, implementation strategy, and potential models of care. Therefore, this study focused on considerations for virtual hospitals in the Australian private hospital context.

Data collection was via semi-structured interviews between February and July 2023. The study design was influenced by both the PERCS Framework and the theory of organisational readiness for change^23,34^.

Primary outcomes: implementation determinants (e.g., barriers and enablers).

Research questions:

1. What are the virtual healthcare needs and preferences of patients with private health insurance and their carers?
2. What are the enablers and barriers to these patients accessing timely and appropriate virtual hospital services at home/in the community?
3. What are the needs, preferences, limitations and perspectives of health and aged care staff who will have a role in delivering, managing, or referring to the virtual hospital?

### Setting and context

In Australian private hospitals, doctors are not hospital staff but independent Visiting Medical Officers (VMOs). There is no financial relationship between VMOs and the health service. Patients are admitted either directly by a VMO or via an emergency centre, under the care of a VMO. Nursing, allied health and auxiliary staff are employed by the hospitals or aged care services. Medical care for inpatients is funded through a variety of sources: federal government Medicare subsidies, private health insurers, Department of Veteran’s Affairs, and self-funded patients. Although Medicare funds in-person care inside a hospital, and some virtual care for discharged patients, there are currently no provisions to fund virtual care for an admitted patient receiving care in the community. Each insurer has an individual contract with the health service, and there may be substantial variance between insurers, e.g., some episodes of care are funded on a per diem basis, whereas others fund on a case payment basis. Some include virtual care, some are silent, and some explicitly exclude it.

## Participants and recruitment

Participants were purposefully selected based on their role using snowball sampling^35^. The research team determined a small initial group of staff and consumers from the health and aged care services to represent a broad set of stakeholders. The initial participant group included health and aged care service executives, internal and external clinicians, and people in other relevant roles such as quality and safety representatives. Informed verbal consent was obtained at the commencement of each interview, which was captured in the audio recording. Participants were located in a variety of areas including metropolitan, regional, and remote areas, so data collection was primarily conducted by video meetings or by phone. At the conclusion of each interview, participants were asked to nominate people they recommended the research team interview and why. The semi-structured interview questions were adapted based on the participant’s role, while the remainder focused on the research questions and PERCS domains^23^. Interviews were conducted by CG (consumers and carers) and OF (staff, VMOs, public health stakeholders). As outlined by Geng, Peiris and Kruk^36^ there is often tension between rigour and relevance in implementation research. There is often a lack of alignment between health service timelines and theoretically ideal research practice, therefore implementation science research employs pragmatic but still robust methods that balance these competing considerations^37^. In this study, because of the broad range of stakeholder groups, to obtain a pragmatic balance between real-world relevancy and research rigour, participant selection and sample size were focused on representation of each group, rather than data saturation. The authors acknowledge that this approach may limit the reliability and generalisability of these results and replication is recommended in other settings.

### Analysis

Interview transcripts were coded by OF (all) and CG (*n* = 27) using a framework analysis method^38^. A-priori codes were derived from the PERCS Framework^23^, with additional codes derived inductively using thematic analysis. Although codes were not explicitly derived from the theory of organisational readiness for change^34^, concepts from this theory were used to inform the interpretation of data. We were particularly interested in whether a shared psychological state of motivation and confidence had been obtained, which would indicate strong readiness for change. Initially, three transcripts were coded by both OF and CG then codes were compared, discrepancies discussed, agreement reached, and the codebook updated accordingly. Throughout the data collection and analysis process, OF and CG discussed emerging findings each week. The strength of each barrier and enabler was rated based on: the frequency with which it was mentioned by participants; the emphasis participants placed upon it; and the potential impact on the virtual hospital. The ratings were agreed by both coders.

### Data storage

All electronic data were stored on secure servers managed by the research institute as per the National Health and Medical Research Council’s Management of Data and Information in Research guide. No paper-based data or consent forms were collected.

### Ethics approval

Ethical approval was received on 9 January 2023 by the UnitingCare Queensland Human Research Ethics Committee, Reference: Fisher_20221207.

## Results

In total, 37 stakeholders participated in 36 interviews, with one husband-wife consumer/carer couple participating in a joint interview. 50 invitations were sent, a 74% participation rate. The largest role group was consumers (*n* = 11, 19·3%), followed by carers (*n* = 7, 12·3%) (Table 1), all of whom were from metropolitan areas. The interviews ranged between 24 and 106 min, with 21 (56·7%) conducted online, six by phone (16·2%) and ten (27·0%) face-to-face.

**Table 1:** Interview participants (*n* = 37).

Sex	Number	Proportion
Male Female	13 24	35.14% 64.86%
Location		
Metropolitan Regional Rural or Remote	33 2 2	89.2% 5.4% 5.4%
Role*		
Allied Health	1	1.75%
Aged Care Community	4	7.02%
Aged Care Residential	6	10.53%
Carer	7	12.28%
Consumer	11	19.30%
Visiting Medical Officer (VMO) or other doctor	6	10.53%
Hospital Leadership	7	12.28%
Nurse	6	10.53%
Health Service Leadership	5	8.77%
Public Health Stakeholder	4	7.02%
Total	57*	100%

*Some participants had more than one role.

**Fig. 1:**
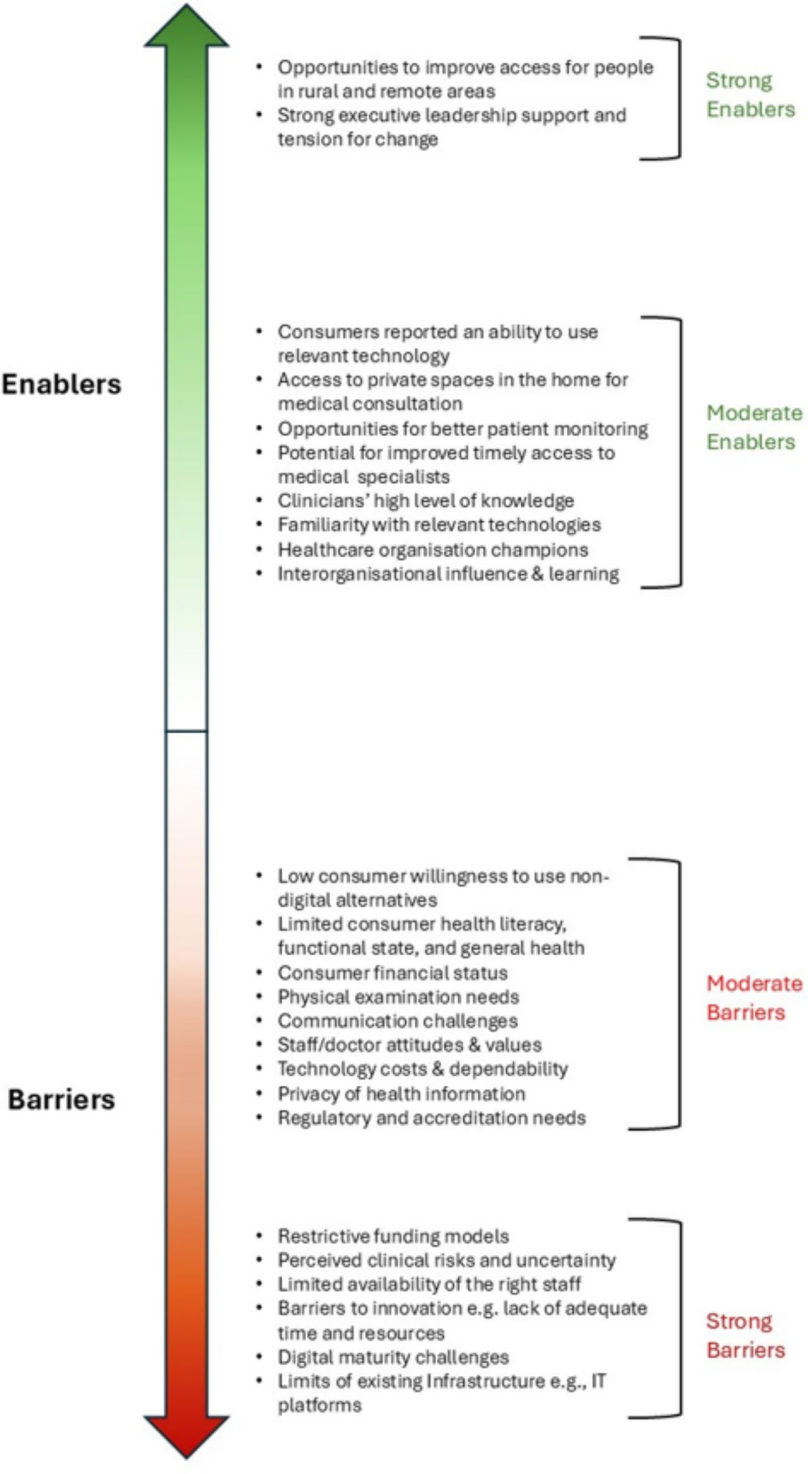
Barriers and Enablers to Virtual Hospital Establishment.

Detailed barriers and enablers are presented in Fig. 1; and Table 2. Overall, seven major implementation determinants emerged:

**Table 2:** Barriers and enablers to establishment of a virtual hospital.

PERCS Domain	Code	Description	Rating	Example Quote/s
The Patient	Ability to use technology	Ability to use technology has increased in the older age group, e.g., the need to use QR codes for daily activities (buying groceries, visiting a doctor) during COVID-19 pandemic.	+ 1	“Interviewer: Are you comfortable using like a laptop or a computer to do a consultation? Consumer: No I hate it. Interviewer: No, you pre-, what do you prefer? Consumer: Face to face… I mean to get scripts and things like that, there are some things that I find it doesn’t matter, but if there is some problem… I want [the doctor] to be able to see me.” “I think particularly we’ve got to be very careful about the elderly because the elderly generations are used to nodding and agreeing. And they’re not like the Gen-X and younger who will tell you, this is a lot of B.S., it’s not helping. They will say, ‘Oh, isn’t this wonderful?’” (Health Service Leadership) “Having Indigenous clients, so with telehealth, we’ve got to think seriously about communication. You… need to be able to see the body of a person who’s expressing their desire to look after you. To them you are reading their body, not just hearing their words. When you come from a custom that is, or a culture which is a verbal culture, not a written culture… you’re reading everything about what that practitioner is actually saying to you.” (Residential Facility Manager) “I’ve seen tele-medicine delivered in some aged care, particularly people with advancing dementia… where it’s just incomprehensible that they would have the television talking to them.” (Residential Facility Manager) “[Health insurance] forms a huge part of our budget… but it’s sort of something I don’t think you can really practically be without.” (Consumer)
	Digital inclusion -diversity of provision	Some consumers preferred phone consultations versus video, and some lacked confidence or comfort using a laptop.	0	
	Digital inclusion - non-digital alternatives	Some consumers were not comfortable with or willing to use technology for healthcare consultations and would always default to using face-to-face services, even if it is less convenient.	-1	
	Attitudes and preferences	Strong agreement across all groups that telehealth must be opt-in not opt-out. Older persons were described as likely to believe what their doctor tells them without questioning, potentially leading to substandard treatment.	0	
	Language and culture	Understanding a patient’s cultural background and preferences was considered essential, particularly for First Nations patients. Communication styles and needs differ between cultures. Practitioners need to adapt communication accordingly.	0	
	Health literacy	There are known challenges for patients navigating health services, and understanding health information, relevant for both face-to-face and virtual care. Practitioners need to actively check patients’ understanding.	-1	
	Functional state	There were differences of opinion about telehealth suitability for people living with dementia or impaired cognitive capacity. Examples of effective dementia models were provided. One doctor stated that virtual hospital patients need the capacity to make a phone call to obtain help.	-1	
	General health	Participants discussed a population trend towards a longer life-span and better physical health for longer, however as people get older cognitive health tends to decline. Concerns were raised about comorbidities and frailty that may impact virtual care suitability. Clinicians considered poor hearing to be a barrier to telehealth.	-1	
	Financial status	Private health insurance can be expensive, and costs need to be considered for people who may have limited budgets. Consumers described private health insurance as a budgetary priority, acknowledging that private health care is a privilege that not everyone can access or maintain.	-1	
The Home and Family	Carer support	The importance of informal/family carers as a critical workforce was raised by multiple participants. For many virtual hospital patients, a carer will be involved, facilitating telehealth consultations, monitoring the patient’s progress, and escalating as required. Carer burden may need to be monitored and addressed. Many carers would prefer to have patients at home where they are more comfortable, less distressed, and at lower risk of perceived hospital-related harms.	0	[Explaining why they would prefer to care for their parent in the home] “Now, when he, if my dad was to come into hospital, he would be disorientated even more, he would be frightened, he would be impulsive, he would get agitated, possibly get aggressive. They’d start to sedate him and use medications, not pain relief, to deal with that behaviour and before I knew it, we would be going down a spiral of drugs that would stop him mobilising, stop him eating.” (Hospital Staff)
	Digital set up and capability	All consumers had a phone. Otherwise, access to technology varied. Some options such as video consulting would not be possible for all patients.	0	
	Geography (e.g., rurality)	Virtual healthcare opens opportunities for people in rural and remote areas. Some participants described a different calculation of risk for patients in rural and remote areas who couldn’t readily access a physical hospital. Telehealth was seen as better than the existing (lack of) options for this group, and potentially worse than the existing options for patients who can readily access a physical hospital.	+ 2	
	Privacy (in the home)	Consumers did not describe any barriers to safety or privacy during healthcare consultations in their home environment.	+ 1	
The Reason for Consulting	Condition or illness	There was a lack of consensus on the types of conditions or illnesses that are appropriate for a virtual hospital. Some of the differences of opinion were influenced by exposure to various virtual healthcare models. One doctor expressed reluctance to have an end-of-life conversation by telehealth.	0	“I, as a fairly traditional physician, I think we underestimate the value of both clinical examination and the value of the healing touch or the hand on the shoulder, the eye contact, the being in the room and feeling the unsaid.” (Doctor) “We run a lot of aged care facilities across the country, some with no access to medical care at all, and I mean that quite literally, and so this provides them with a potential opportunity to get medical care that they may not otherwise get.” (Health Service Leadership) “I’ve seen errors in telehealth, I’ve seen doctors who have done telehealth assessments on older people, where nobody’s examined that person. They’ve missed pneumonia, they’ve missed kidney stones, they’ve missed a bowel obstruction, they’ve missed an ischemic valve, they’ve missed [a myocardial infarction]. You know, all because they think they can do all these things on telehealth, because they’ve not partnered it with a very clear idea of, well, these are the things I can do on telehealth, and these are the things that an older person needs to have done in person.” (Public Health Stakeholder)
	Physical examination needed	There was agreement that many types of assessment require physical contact. However, some could be conducted using hybrid models with a practitioner or carer face-to-face with the patient, and a doctor via telehealth. The importance of physical contact or “healing touch” as part of the clinical consultation was raised.	-1	
	Reason clinician wants to see patient	Monitoring of a person’s health status was of key interest for clinicians.	+ 1	
	Reason patient wants to be seen	Consumers reported that there are few gaps in the private health system for those who live in hospital catchment areas and have the ability to pay for private healthcare. Consumers and carers want timely access to specialists.	+ 1	
	Reason aged care wants patient seen^#^	Residential aged care facility participants reported that the main attraction of the virtual hospital for them is to obtain timely medical assessment and advice.	+ 1	
	Reason health service wants patient seen^#^	Business growth was a strong priority for health service leadership participants.	+ 1	
	Risk, trajectory, uncertainty	Concerns were raised about missing clinically important signs during remote assessment, potentially leading to misdiagnosis, inappropriate treatment, or adverse outcomes. Participants acknowledged that some patients are harmed in hospital, e.g., hospital acquired infections, and these harms need to be weighed against the risks of a home environment which the hospital cannot control.	-2	
	Urgency and severity	Triage was seen as very important. Time-critical conditions were considered unsuitable for the virtual hospital.	0	
The Clinical Relationship	Communication	Face-to-face communication was preferred by both consumers and clinicians. Clinicians’ English fluency and accents were important considerations for consumers when using telehealth. The ability of staff to speak in local dialects with First Nations patients was raised.	-1	“My biggest lesson from all this is that… telehealth plays a very small part. The power of being present with an experienced clinician… [experienced nurses are] excellent at the physical examination and assessment of an older person. They can be my eyes and ears. So, I can have my video telehealth assessment, but I need to trust the hands at the other end, and that person being my best practitioner is how I do that.” (Doctor)
	Trust	Creation of trust was considered important by all groups, both for encouraging patients to engage in telehealth, and during the consultation. A key element of trust was the privacy of their information and understanding who is “on the other end,” i.e., who is conducting and witnessing the consultation.	0	
Staff and Doctors	Attitudes, values	Healthcare providers were seen as generally driven to provide high quality patient care. Conversely, some staff were seen as wanting to just do their job and leave. Anxieties were raised, including issues of trust between businesses and disciplines. Some participants expressed ageism or incorrect assumptions about older people. In particular, there was a common assumption amongst participants that older people didn’t know how to use technology. Participants stated not all staff would welcome a move to virtual healthcare delivery, preferring the traditional face-to-face model. ‘Ownership’ of patients was an issue, and some hospital staff were sceptical about the quality of care if they refer ‘their patients’ into the service.	-1	“Not that we don’t trust anyone, but we don’t trust anyone, yeah, so we’ll just go in and do our own assessment.” (Aged Care Staff) “One of my beefs about ageing people and illness and medical care is that sometimes people just put… the older patients on the shelf a little bit and say oh you’ll be right.” (Consumer) “So, I think it is a conceptual mind shift for clinicians and people do need to be trained in how to do it… I think it’s overcoming some of those kind of urban myths around virtual care, telehealth for, for clinicians who have not traditionally been working in that model.” (Health Service Leadership) “The change fatigue… I think is huge, I think people are surveyed out, I think they’ve had enough… you’re not going to get buy-in, ‘cause people are sick of it, you know, they don’t want to hear it, it’s just one thing on top of another.” (Health Service Leadership) “I think we’re… pretty adaptable and they’re pretty, pretty keen to change and they’re pretty enthusiastic. So, I think, I don’t think they’re particularly fatigued. I think we’ve come out the other side of that… I think they’re pretty much back to, get back to the future and just getting on with it.” (Hospital Staff)
	Availability of the right staff	Workforce issues exist across the Australian health and aged care sectors. Aged care and hospital participants acknowledged that staff shortages and retention challenges require new and more efficient ways of working. Residential aged care staff raised concerns about the potential burden on their staff that might be created if they are needed to support virtual consultations for residents. Telehealth roles were seen as attractive to staff wanting a change in role.	-2	
	Knowledge, capabilities	Although staff in all groups were seen as capable and knowledgeable in their own areas, being able to access timely, interdisciplinary support was considered a useful backup to existing processes. A strong learning culture was described. Training and education on telehealth strategies was considered essential.	+ 1	
	Vulnerability and risk	There was a perception from some participants that staff are fatigued from years of continual, usually top-down changes to their ways of working. Staff are expected to adapt, which creates risks to staff (fatigue), and to the virtual hospital through lack of engagement. In contrast, other participants felt that staff were past the fatigue prevalent towards the end of COVID-19 restrictions.	0	
Technology	Cost	Participants commented on the importance of upfront investment in development of technology solutions that are purpose-built, adaptable, and able to be maintained and built upon. This was expected to be costly.	-1	“Two clinicians wanted to quit the other week because… they’re there in front of the patient and they couldn’t log on.” (Hospital Staff) “It has to be easy, functional and work… if it doesn’t it just gets put in the cupboard and never seen again.” (Aged Care Leadership) “There’s different packages of, software packages that don’t always talk with each other, because it’s been in place for x number of years as well the ability to talk to each other has become obsolete in some areas because as you upgrade and update one package it stops talking to another package” (Hospital Staff)
	Dependability	Participants raised concerns about the dependability of the technology required to run the virtual hospital. Examples from the physical hospitals included system crashes, slow logins, and impaired internet access at peak times.	-1	
	Familiarity	Since the COVID-19 pandemic, participants across all groups reported they are more comfortable and familiar with video conferencing and telehealth.	+ 1	
	Functionality	There was strong agreement from participants that the virtual hospital would need an electronic medical record accessible in real time by all hospital staff. Healthcare providers expressed the critical importance of technology that communicates in real time with existing hospital and aged care databases.	0	
	Maintenance and upgrades^#^	Participants stressed the importance of maintenance and ongoing upgrades of the electronic medical record and other technologies which would enable the virtual hospital to grow.	0	
	Privacy of health information^#^	One consumer had concerns about the information that would be collected and stored within an app. They did not want any of their sensitive health information stored in an app.	-1	
The Healthcare Organisation	Access policies	The health and aged care services sectors are difficult to navigate, and care navigation needs were raised by all groups.	0	“So for me it’s firstly a sustainability measure, secondly patient demand and third delivering to places that we haven’t delivered before, which may not even be in Australia, it could be internationally as well.” (Health Service Leadership) “The hospital system’s designed around doctors. If you think about how everything works, it’s essentially going back to the Florence Nightingale type approach… The care has progressed, but the structure of the system hasn’t changed in a hundred years, and it revolves around ‘the doctor will see you now’ model, rather than actually what’s right for the patient, and we need a bit of a mindset shift from that perspective.” (Health Service Leadership) “I think you’d have to be careful getting doctors offside… if you did look like you were competition.” (Hospital Leadership) “You’ve got to have the right person in the job to build that trust, and communication is a key element of that and then, you know, delivering on what you say you’re going to do is really critical for the, for the doctors, yep.” (Health Service Leadership) “It’s a different way of working so that’s always that big change management piece around what’s the why, what’s the narrative around why we actually need to change what we do. So again, it comes back to being really clear about what our remit is and what it is that we intend to do so that we can have a very clear narrative for the stakeholders about why we want them to change the way that they work or what they do.” (Health Service Leadership)
	Innovativeness and culture	Although many participants were welcoming of and enthusiastic about change, they noted the need for pragmatic planning, in particular, bringing staff on-board prior to implementation. Barriers to innovation were raised, including: - Organisational siloes - Funding models that stifle creativity and adaptation - Lack of adequate slack for creativity, i.e., staff are working to their capacity without time to design and implement new innovations.	-2	
	Level of digital maturity	Participants described willingness but limited current capacity to transition to digital workflows across the organisation. Participants stated this transition would require substantial investment, training, and changes to work practices. Concerns were raised about the extent to which some stakeholders, such as VMOs, would be willing to transition from paper-based practices to adopt new technologies.	-2	
	Level of digital maturity - capability	Participants identified opportunities to expand existing telehealth services.	+ 2	
	Level of digital maturity - infrastructure	Some participants had issues logging on to a Teams call or speaking over mobile phone during their interview due to connectivity issues. These issues would also impact capacity for virtual care provision.	-1	
	Readiness	Participants expressed the importance of having a clear virtual healthcare strategy. This was considered critical to ensure readiness for implementation of a virtual hospital.	0	
	Readiness – executive leadership support	Strong executive leadership support was described. Virtual healthcare was seen as necessary for business sustainability, as well as an opportunity to address patient preferences.	+ 2	
	Readiness – innovation-system fit	The system-fit was not clear at the time of data collection because the model had not yet been defined. There was strong support for creating a virtual hospital designed around the needs and preferences patients rather than doctors, however it was noted that this would potentially require a cultural shift.	0	
	Readiness - opponents	Many participants expressed concerns about how the hospital might impact VMOs and other businesses. There was strong opposition to any model that would, e.g., take work away from VMOs, or target the same patients as existing services.	-1	
	Champions	Examples were given of previous successful innovations. Strategies that had worked previously included: engaging staff champions and project facilitators at a local level, clear and regular communication with frontline staff, developing trusting relationships with stakeholders, and responding to complaints and feedback in a timely and effective manner.	+ 1	
	Tension for Change	Health and aged care providers expressed strong tension for change at an organisational level, however there were differences of opinion about whether individual VMOs and staff felt that same tension.	+ 2	
	Systems and logistics	Having clear, consistent clinical governance structures was seen as crucial for patients, clinical providers, and the healthcare organisation.	0	
	Workload and resources	Workloads are busy across all staff groups, in part related to workforce issues. This impacts capacity to take on training or additional tasks.	-1	
	Unintended Consequences^#^	Patient redirection to the virtual hospital may reduce patient numbers for other services. Hospital avoidance, although a strong driver for public health stakeholders, was not seen as attractive from a business perspective for private hospital stakeholders and VMOs.	-1	
The Wider System	Infrastructure	The lack of technological infrastructure allowing real-time digital communication and sharing of patient information was described as a major barrier. This related both internally, and externally with ambulance, public hospitals, external aged care providers, and general practitioners. Streamlining real-time patient communication was considered a necessary precursor to a virtual hospital by some stakeholders.	-2	“I’m having to review the patient every day virtually but there’s no funding mechanism for me to be paid to do that work, whereas when the patient’s sitting in a hospital bed, I actually do get remunerated for the work that I do, and I don’t think it’s an unreasonable expectation for people to be remunerated for the work that they do… You’re still having to provide that clinical care and the clinical governance and be accountable and responsible for that patient’s care but you’re not being remunerated for it, and I think that’s a big barrier.” (Doctor) “The bit that [health insurers] worry about is once you move those people into the community you’ve now got more beds to admit people to so in fact their overall spend goes up. So, unless you close beds what they see is that, well, this hospital has 500 beds and you’ve now got 50 outpatient beds, you’ve now gone from a 500 bed to a 550-bed hospital and in real terms for them that costs more dollars.” (UnitingCare Leadership) “Interviewer: Is there anything missing in your current healthcare ? Consumer: No. Interviewer: No, you don’t feel like there’s any… Consumer: No. Interviewer: …gaps or anything? Consumer: Not at all, not at all.”
	Interorganisational influence and learning	Public health stakeholders were open about their learnings from developing virtual health services, both to ensure minimal overlap, and to enable private patients to be directed into the private system as appropriate.	+ 1	
	Policy and regulatory issues	The complexity of developing a standalone hospital was raised, particularly the need for hospital accreditation.	-1	
	Funding sources and limitations^#^	Health service stakeholders raised funding issues as a major barrier. This was a consistent theme across all health service stakeholder data. There was a perception that current funding models, both from Medicare and private insurers, do not support innovative models of virtual healthcare delivery. Key concerns included: - Medicare rebates are not available for virtual hospital patient consultations, so there was a lack of clarity about payment of VMOs. - Concerns that a virtual hospital inpatient cannot access other government-funded services. - Developing a standalone virtual hospital requires contract negotiations with health insurers.	-2	
	Availability of health services	The major gap in health services raised across all groups was timely access to general practitioners. This means that important preventative care or management of minor health issues may be delayed, risking the development of more complex long-term health issues. Otherwise, consumers reported that there were no gaps in health services accessible to them in the private health sector.	0	
	Public versus Private^#^	Consumers felt that there were substantial differences between the public and private systems. There was agreement across participant groups that private patients often receive care more quickly than public patients.	0	

# Inductively derived code, not present in PERCS Framework. Rating scale: +2 = Strong enabler; +1 = Enabler; 0 = Neutral; -1 = Barrier; -2 = Strong barrier.

1. Opportunities from removing geographical boundaries;
2. Challenges with technology and digital readiness;
3. Differences in consumer and clinician perceptions about telehealth suitability and acceptability;
4. Virtual healthcare funding barriers;
5. Concerns about implementation process;
6. Internal and external communication challenges;
7. Workforce opportunities and issues.

### Removing geographical boundaries

Participants from all groups expressed excitement about virtual healthcare possibilities, particularly improving access to specialist healthcare services for people living in rural and remote areas. There was strong agreement that the future of healthcare is digitally enabled. The desire to provide the best possible care for patients, in the environment that is right for the patient, was a strong theme across all data. From the consumer and carer perspective, hospitals provide much more than just acute admissions – for example, consumers visit specialist doctors, attend day services such as rehabilitation, and have diagnostic testing. Consumers, carers and clinicians encouraged the healthcare service to be aspirational and build a virtual hospital that incorporates, where possible, the same broad range of services that patients access in physical hospitals.

### Technology and digital readiness

A virtual hospital, by its nature, is system oriented. However, there are substantial barriers to achieving a system-oriented culture as outlined by the PERCS scale of digital maturity for healthcare organisations^23^. There is minimal opportunity at present for data exchange between private hospitals, public hospitals, and aged care providers. However, stakeholders felt strongly that developing more streamlined real-time internal and external communication about patient status and key clinical information was necessary for the success of the virtual hospital.

### Telehealth suitability and acceptability

There was a lack of agreement amongst participants about the suitability and acceptability of virtual healthcare. Some were very excited about the possibilities, and identified advantages such as reduced risk of hospital acquired infection. Others expressed concerns about the risks of inadequate clinical assessment leading to potential misdiagnoses and harms to patients. Consumers and carers expressed a strong preference for face-to-face consultations, unless their needs were simple, e.g., a repeat prescription from a known provider.

### Funding

Currently, for the most part, private health insurer and Medicare funding models are restrictive, and do not enable virtual healthcare innovation. This was a very strong theme across all data. Without changes to health fund agreements and Medicare funding models, e.g., to enable payment of VMOs who provide medical care to a patient in a virtual hospital ward, the potential models of care are limited. Although some VMOs have expressed willingness on occasion to oversee private patients in a virtual ward without accessing Medicare fees if the care provided would be more suitable for a patient, this was seen as an unreasonable and unsustainable imposition on VMOs.

### Implementation process

Participants reported they needed to understand the vision, scope, and purpose for the Virtual Hospital. Concerns were raised about the potential for rushed implementation without sufficient planning and consultation. This was seen as common in large healthcare providers.

### Communication

Communication issues, both internal and external, were a strong theme across all healthcare staff. A reported lack of certainty about how the virtual hospital may impact their patients and businesses led participants to express anxiety and scepticism.

### Workforce

Although healthcare staff were described as highly skilled in face-to-face healthcare provision, training will be required to support their adaptation to virtual healthcare provision. Workforce issues across the health and aged care sectors were described as barriers, with challenges for recruitment and retention. However, virtual healthcare offers opportunities to re-engage an existing workforce, e.g., experienced practitioners who are no longer able to provide face-to-face care, such as those with a history of injury.

## Discussion

This study identified a comprehensive list of barriers, enablers and considerations for design and implementation of a virtual hospital, and demonstrated the importance of using evidence-based implementation theories, frameworks and processes in the pre-implementation phase. Seven major implementation determinants and 53 considerations were identified (Table 2). The broad range of identified determinants will enable the health service to make crucial adaptations to improve the likelihood of suitability, acceptability and uptake of the virtual hospital. The need for a long-term vision and clear principles for the virtual hospital was a strong message. This research addressed critical implementation research gaps^12,13^.

The major barriers identified, for the most part, aligned with previous research, with some notable differences. Australian private hospital funding mechanisms are currently restrictive, representing a critical barrier to establishment of private virtual hospitals. This is a novel finding not represented in previous literature. At the time of writing, the Australian Government’s Health Insurance Act^39^ Health Insurance Determination 2021 allows Medicare Benefits Services payments for a specialist doctor to conduct a face-to-face consultation with an admitted patient, or a telehealth consultation with an outpatient, but not a telehealth consultation with an admitted patient. This funding restriction has substantially impacted the viability of private virtual hospitals in Australia. This finding is broadly in alignment with previous research in the Australian public hospital system^27^. In both sectors, funding models were found to stifle virtual healthcare innovation. There was also alignment with previous studies on the need for a clear vision and purpose for the virtual hospital^27^, strong governance structures and clinical processes^27,28^, safety concerns about virtual healthcare^21^, the importance of trust and relationships with key stakeholders^7,27,40^, workforce challenges and training needs^27^, and technological limitations and challenges^7,27^. These are consistent findings across varied settings, including public and private hospitals and different countries, indicating that these determinants are important to consider in virtual hospital planning, acknowledging that there may be differences between contexts.

Differences between clinicians’ perceptions of virtual healthcare suitability and acceptability were found between studies. In our study, clinicians’ perceptions of virtual healthcare suitability differed for various conditions. These perceptions appeared to be influenced in part by exposure to virtual healthcare models: for the most part, greater exposure related to higher perceived suitability. Schultz et al. reported clinicians in their study had a high level of acceptance of virtual COVID-19 care overall^7^. By contrast, Shuldiner et al. found that clinicians’ perceptions of the suitability of virtual healthcare varied^18^, and this was a major influence on whether they had normalised virtual healthcare. These discrepancies highlight the need for further exposure of clinicians to virtual healthcare models, additional training in virtual consultation practices, and further research to understand in more detail how clinicians’ attitudes towards virtual healthcare vary across disciplines, settings and levels of exposure. Based on Shuldiner et al.’s findings it will be important to ensure that clinicians working in a virtual hospital perceive the value of their work and feel that they are able to operate at their full scope of practice. Although there were differences on some other points between participants, e.g., the drivers and tension for change at an individual level, there were no substantial differences in responses between disciplines or types of stakeholders. Where there were minor differences of opinion, these have been noted in Table 2.

Consumers and carers expressed a strong preference for face-to-face healthcare over telehealth, although they acknowledged that these perceptions had changed to some extent during the COVID-19 pandemic. This reflects a large body of research on the perceived acceptability of telehealth (e.g.,^41,42^). Strategies such as care navigation can be effective in improving the perceived acceptability of telehealth modalities^43^, and it may be necessary to consider incorporation of a care navigation or concierge service within the new virtual hospital. Another novel finding raised by participants was the opportunity to maintain or re-engage a clinical workforce who may not wish to or cannot continue traditional face-to-face clinical care, e.g., due to an injury. The flexibility with hours and locations that virtual hospital care offers was seen as attractive to clinicians.

From an organisational readiness to change perspective, although there was substantial goodwill and motivation for change, there was a lack of confidence due to technical and funding barriers, and a lack of understanding of the overarching vision of the virtual hospital. Participants from all groups expressed concerns about the risks of virtual consultation and the potential for misdiagnosis or inappropriate treatment prescription. Interestingly, participants reported differences in their risk calculation for people living in metropolitan versus rural and remote areas. In Queensland, Australia there are vast geographical distances between rural and remote population centres and a consumer may be multiple days driving distance from their closest tertiary hospital, therefore timely access to medical care is lacking. Where a patient lived near a physical hospital, the expressed preference was for face-to-face healthcare, with virtual healthcare considered substandard. However, for patients unable to access a physical hospital in a timely manner, a virtual assessment was considered by participants to be appropriate, and an improvement on the current lack of timely healthcare options. This indicates a tacit acceptance of a lower standard of care based on circumstance. This is a novel finding, and we have expanded on this in a separate manuscript^44^. This discrepancy between the expected care standard in metropolitan versus rural areas highlights known healthcare inequities experienced by people in rural areas^45^.

Providing healthcare ‘in place’ through virtual mechanisms aligns with the global push for ageing ‘in place:’ providing necessary care and supports in the community to enable older people to remain in their home environment, connected to social and community networks.^46^ Consumers reported their high budgetary priority of private health insurance because it enabled them to access specialist doctors in a timely manner. This willingness to pay more to access specialists aligns with Vo et al.’s review of discrete choice experiments eliciting patients’, healthcare providers’ and policymakers’ preferences and willingness to pay for virtual care.^47^

With its emphasis on remote consultation specifically, the PERCS Framework provided an appropriate set of domains for planning of a virtual hospital^23^. Telehealth consultation brings with it specific risks, challenges, and advantages that differ from the implementation of other types of clinical services. Having admitted inpatients being clinically treated in their home or community increases the complexity of virtual hospitals. PERCS compared favourably for this study with the Nonadoption, Abandonment, Scale up, Spread and Sustainability (NASSS) Framework^33^, which is commonly used to guide the implementation of technological innovations, and the Consolidated Framework for Implementation Research^48^, a determinants framework commonly used when conducting a context assessment. PERCS provided a more suitable guide for this study due to the explicit inclusion of constructs focusing on the home environment and the clinical consultation. It is common practice in implementation science research to adapt and combine theoretical approaches and frameworks to suit the context of individual projects. In this case, the framework required adaptation to suit the Australian private hospital context by adding VMOs as a stakeholder group, and having a greater focus on funding models. Additional research is necessary to understand the safety and effectiveness of virtual hospital models. It will be important to evaluate new virtual hospital models using a hybrid implementation-effectiveness design to determine clinical, service and implementation outcomes for patients, carers, staff, VMOs and the healthcare service. The perspective of informal carers, including the burden that caring for an inpatient in their home may represent for informal carers, is a critical gap in existing literature. This is an important area for future research, as informal carers are likely to be a key healthcare workforce for many virtual hospital models of care.

### Implications for policymakers, clinicians, healthcare decision makers and health insurance providers

The major barriers identified in this study related to government and health insurance funding models. The authors encourage Australian policymakers to consider amending legislation that currently restricts the payment of Medicare Benefits Scheme item numbers for medical consultation by telehealth for admitted patients. Likewise, the authors encourage policymakers in other countries to consider how existing funding models may stifle virtual hospital innovation. For healthcare decision makers planning to implement a virtual hospital, Table 2 provides a useful guide to barriers and enablers for consideration. Pre-implementation virtual hospital research needs to be complemented with tailored implementation strategies to address the identified barriers and enablers, and evaluation of implementation outcomes. This should be a focus for future research. Technological interconnectivity, accessibility, reliability and internet access are critical for a virtual hospital but not currently a reality, particularly for people living outside of major Australian cities. This was witnessed during interviews where video conferencing software experienced multiple lags and outages that influenced the flow of conversation and created delays. Australian governments need to invest in higher speed and more reliable internet services across Australia to enable efficient, reliable and safe virtual hospitals.

### Strengths and limitations

This study addresses an important gap in knowledge by outlining a comprehensive set of critical barriers and enablers to the establishment of a virtual hospital. The study used a novel, evidence-based, multi-theoretical implementation science approach which enabled a broad range of barriers and enablers to be identified. There was strong interest from participants, with the majority of invitees agreeing to an interview. Although this study was specific to the Australian private hospital context, the list of considerations is relevant internationally in both public and private settings, noting that the specific determinants may differ based on context. There is a risk in all qualitative studies that the interpretation of data may be influenced by the perspectives and biases of the researchers. We have used reflexivity strategies to minimise the impact of any biases, such as having multiple coders, and an inclusive multi-stakeholder research team who all contributed to the interpretation of data. The decision to focus on role representation rather than saturation also limits the generalisability of these results. Additional participant groups not included in the scope of this study may need to be consulted. These include general practitioners and other stakeholders who are likely to refer patients to the virtual hospital, a more diverse consumer group, including consumers and carers from rural and remote areas, First Nations peoples, and clinical staff who will be directly providing remote consultations. We collected limited demographic data from participants and acknowledge that additional demographic information such as age and ethnicity may better contextualise these results. All consumers were current patients or clients of the health service which means their responses may be influenced by their relationship with the health service. Therefore, caution must be used in the interpretation of these data, as they will not be representative of a broader health service user population.

## Conclusion

Using an implementation-science design enabled the identification of a broad range of barriers and enablers to establishment of a virtual hospital in the Australian private hospital context. This set of considerations has international relevance as public and private virtual hospitals are rapidly established. This research makes a unique contribution to the virtual hospital literature by addressing critical evidence gaps on the implementation determinants for virtual hospitals, and specific considerations that are relevant in the private hospital sector. The PERCS Framework provided an appropriate guide to domains for consideration when planning a virtual hospital, with some minor adaptations to suit the context of this study. Further research is needed to determine the influence that these barriers and enablers have on the effectiveness of this and other virtual hospitals. Although there was strong tension for change from business sustainability and patient-centred care perspectives, substantial barriers to establishment of a private virtual hospital were identified. At the time of writing, the major barrier was government and health insurance provider funding models, particularly the inability of a VMO to access Medicare Benefits Scheme funding for a telehealth consultation with admitted patients. Many of the barriers identified are potentially malleable, although some, such as federal government funding models, may be outside of the sphere of direct influence of the healthcare provider.

## Data Availability

The data for this study will not be shared as it is potentially identifiable, and we do not have permission from the participants or ethics approval to do so. Transcripts clearly identify the roles and responsibilities of each participant throughout, both directly and indirectly, and are not able to be de-identified. The coding dictionary is available on request of the corresponding author.
